# Restrictive hypertonic saline resuscitation is associated with less intestinal injury after hemorrhagic shock in rats and with coordinated changes in MALAT1, miR-145-5p, and BNIP3 expression

**DOI:** 10.3389/fsurg.2026.1920366

**Published:** 2026-09-30

**Authors:** Hongfei Ma, Yang Zhang, Jie Yang, Tianshan Wu, Gongbing Zhu, Xinwen Yang

**Affiliations:** The First Affiliated Hospital of Xinjiang Medical University, Urumqi, Xinjiang Uygur Autonomous Region, China

**Keywords:** BNIP3, hemorrhagic shock, hypertonic saline, intestinal ischemia-reperfusion, MALAT1, miR-145-5p, restrictive resuscitation

## Abstract

**Background:**

Intestinal ischemia-reperfusion contributes to barrier failure and secondary organ injury after hemorrhagic shock. It is not known whether restrictive hypertonic saline resuscitation alters the resulting intestinal injury or the accompanying non-coding RNA response.

**Methods:**

One hundred male Sprague–Dawley rats were allocated to normal control, hemorrhagic shock, conventional 0.9% saline resuscitation (CFR), restrictive 7.5% hypertonic saline resuscitation (RFR), or superior mesenteric artery ischemia-reperfusion control groups. Hemorrhagic shock was induced by controlled blood withdrawal to maintain mean arterial pressure (MAP) at 35−40 mmHg for 60 min. CFR targeted an MAP of approximately 80 mmHg, whereas RFR targeted an MAP of approximately 60 mmHg. Three-hour survival was recorded. Coagulation, serum IL-6 and TNF-α, qRT-PCR, Western blotting, H&E staining, and TUNEL were assessed in assay-specific subsets. Public GEO datasets were analyzed for exploratory transcriptomic context.

**Results:**

Three-hour survival was 75% in RFR and 60% in CFR (two-sided Fisher exact test, *P* = 0.501). In the coagulation cohort, prothrombin time (PT) and thrombin time (TT) were shorter in RFR than in CFR after Dunn–Holm adjustment; no differences were observed for activated partial thromboplastin time (APTT), international normalized ratio (INR), and prothrombin activity (PA). RFR had lower serum IL-6 and TNF-α, better-preserved ileal morphology, and lower TUNEL positivity in representative fields than CFR. MALAT1 was lower, while miR-145-5p and Bnip3/BNIP3 were higher in RFR. Exploratory analyses of public datasets confirmed that Malat1 and Bnip3 are expressed in the intestinal cell populations relevant to ischemia-reperfusion injury.

**Conclusion:**

Under the present experimental conditions, restrictive hypertonic saline resuscitation was associated with a less severe acute intestinal injury phenotype than conventional isotonic resuscitation. The coordinated changes in MALAT1, miR-145-5p, and BNIP3 do not establish a linear regulatory axis and require functional validation.

## 1 Introduction

The intestine is among the organs least tolerant to hemorrhagic shock. Villus epithelial cells have a high metabolic demand and rely on a countercurrent villus microcirculation, making the mucosa vulnerable to an early reduction in oxygen delivery during hypoperfusion and delayed recovery after reperfusion. The ischemic mucosa loses tight-junction integrity, and translocation of luminal antigens and inflammatory mediators converts a local barrier lesion into a systemic inflammatory response (1, 2). Because this sequence is initiated by the shock state and amplified by reperfusion, gut-derived injury contributes substantially to organ dysfunction and late mortality following otherwise successful resuscitation after trauma (3, 4).

Fluid resuscitation is a crucial component of hemorrhagic shock management, but the optimal strategy remains debated. Large-volume isotonic crystalloid quickly restores macrocirculatory pressure; however, it also dilutes coagulation factors, increases hydrostatic load, and aggravates tissue edema. Restrictive and permissive-hypotension approaches aim to preserve essential perfusion while minimizing these effects (5, 6). Hypertonic saline addresses the issue differently. A small infused volume creates a crystalloid osmotic gradient that shifts interstitial and intracellular water into the vascular compartment, restoring preload without a comparable hydrostatic burden. The accompanying reduction in endothelial and erythrocyte swelling can widen capillary lumina in the mucosal microcirculation. Hypertonic exposure has also been reported to blunt neutrophil activation and adhesion. It remains unclear whether the injured intestine responds differently, both structurally and molecularly, to restrictive hypertonic resuscitation compared to conventional isotonic resuscitation.

Non-coding RNAs provide a way to approach this question. Long non-coding RNAs regulate gene expression through chromatin, transcriptional, and competing endogenous RNA mechanisms (7, 8). MALAT1 has been linked to ischemia-reperfusion injury, endothelial activation, and cell survival in several organs (9–12), while miR-145-5p is associated with tissue repair and autophagy-related pathways (13). BNIP3 is a hypoxia-inducible BCL2-family protein with two potential outcomes in ischemic tissue: It can trigger regulated cell death or promote mitophagy and mitochondrial quality control (14, 15). It is still unresolved which outcome predominates after intestinal ischemia-reperfusion, and this understanding is crucial for interpreting changes in BNIP3 expression.

We therefore investigated whether restrictive hypertonic saline resuscitation, compared with conventional isotonic resuscitation, is associated with differences in acute survival, coagulation, systemic inflammation, ileal morphology, TUNEL positivity, and MALAT1/miR-145-5p/BNIP3 expression after hemorrhagic shock. Bulk and single-cell transcriptomic analyses of public ischemia-reperfusion datasets were included for biological context, rather than to establish regulatory causality.

## 2 Materials and methods

### 2.1 Ethics

Procedures were approved by the Institutional Animal Care and Use Committee of Xinjiang Medical University (approval number: No. IACUC-JT-20230515-08) and conducted in accordance with the National Institutes of Health Guide for the Care and Use of Laboratory Animals.

### 2.2 Animals, group allocation, and experimental batches

One hundred male Sprague–Dawley rats (220−250 g) were housed under standard conditions (22 ± 1 °C, 50%–60% humidity, 12-h light/dark cycle) with free access to water. Food was withheld for 8 h before the experiment. The same fasting period was applied to every group to reduce variability related to recent food intake. Fasting can alter the basal metabolic and morphological state of the intestinal mucosa; because the exposure was standardized, we treat it as a common background condition and address it in the limitations.

Animals were divided into five groups: normal control (NC) (*n* = 10), hemorrhagic shock (HS) (*n* = 20), conventional 0.9% saline resuscitation (CFR) (*n* = 20), restrictive 7.5% hypertonic saline resuscitation (RFR) (*n* = 20), and superior mesenteric artery ischemia-reperfusion control (SEC) (*n* = 30; 15-, 30-, and 45-min superior mesenteric artery occlusion subgroups, *n* = 10 each). The work was carried out in two batches. Batch 1 included 36 rats (NC, *n* = 4; HS, *n* = 8; CFR, *n* = 8; RFR, *n* = 8; SEC, *n* = 8) and Batch 2 included 64 rats (NC, *n* = 6; HS, *n* = 12; CFR, *n* = 12; RFR, *n* = 12; SEC, *n* = 22). Assay-specific subsets were drawn from the original 100 animals based on planned measurements and specimen availability; the biological sample size for each assay is explicitly stated in the relevant Methods section, figure legend, or Supplementary Table. The experimental design, animal allocation, and study assessments are summarized in Figure 1.

**Figure 1:**
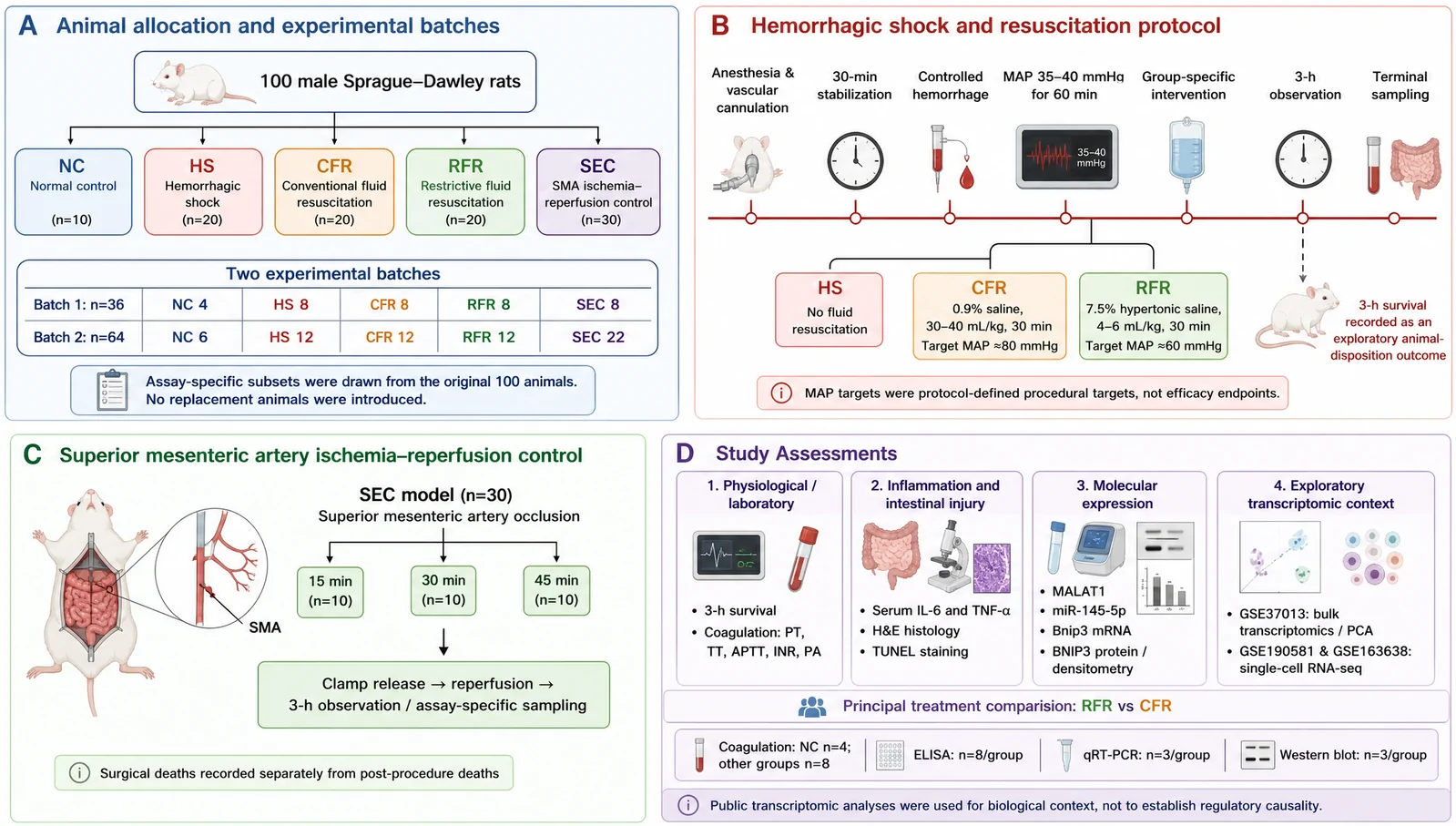
Experimental design, animal allocation, and study assessments. One hundred male Sprague–Dawley rats were allocated to NC (*n* = 10), HS (*n* = 20), CFR (*n* = 20), RFR (*n* = 20), and SEC (*n* = 30) and studied in two batches (36 and 64 animals), with assay-specific subsets drawn from the original 100 animals; no replacement animals were introduced. Hemorrhagic shock was established by maintaining MAP at 35−40 mmHg for 60 min. CFR received 0.9% saline over 30 min to achieve a target MAP of approximately 80 mmHg; RFR received 7.5% hypertonic saline over 30 min to achieve a target MAP of approximately 60 mmHg. These MAP values were procedural resuscitation targets, not efficacy endpoints. Three-hour survival was recorded separately. Assessments comprised coagulation profiling, IL-6/TNF-α ELISA, qRT-PCR of MALAT1/miR-145-5p/Bnip3, BNIP3 Western blotting, H&E histology, TUNEL staining, and exploratory GEO analyses. MAP, mean arterial pressure; NC, normal control; HS, hemorrhagic shock; CFR, conventional fluid resuscitation; RFR, restrictive fluid resuscitation; SEC, superior mesenteric artery ischemia-reperfusion control. (**A**) animal allocation and experimental batches; (**B**) hemorrhagic shock and resuscitation protocol; (**C**) superior mesenteric artery ischemia–reperfusion control; (**D**) study assessments.

No animals beyond the original 100 were introduced as replacements. Procedural failures and deaths were recorded in the animal disposition record. Terminal tissue and molecular analyses necessarily reflect animals from which appropriate specimens could be obtained, while deaths were included in the 3-h survival record.

### 2.3 Hemorrhagic shock model, success criteria, resuscitation, and survival observation

Rats were anesthetized with 1% pentobarbital sodium (1 mL/100 g, intraperitoneally) and placed supine on a thermostatic heating pad. Body temperature was maintained at 36.5°C−37.5°C. The left carotid artery was cannulated for controlled blood withdrawal and continuous mean arterial pressure (MAP) monitoring, and the tail vein was cannulated for fluid administration. Catheters were flushed with heparinized saline (10,000 U/L). After a 30-min stabilization period, 500 U heparin was administered through the arterial catheter.

Successful establishment of hemorrhagic shock was defined as successful vascular cannulation with stable hemodynamic monitoring, reduction of MAP to 35−40 mmHg by controlled blood withdrawal, and maintenance of MAP within this range for 60 min through further withdrawal or reinfusion as required. Failure to reach or maintain the target MAP due to a procedural issue was classified as unsuccessful model establishment, not as a treatment outcome.

After the shock phase, HS animals received no resuscitation fluid. CFR animals received 0.9% saline over 30 min, titrated to achieve MAP of approximately 80 mmHg, at a volume of approximately 30−40 mL/kg. RFR animals received 7.5% hypertonic saline over 30 min, titrated to achieve MAP of approximately 60 mmHg, at an administered volume of approximately 4−6 mL/kg. The shed blood volume needed to maintain shock MAP was approximately 18−22 mL/kg. Because treatment was MAP-guided, final blood loss and infusion volumes varied among animals. The MAP values reached after CFR and RFR were protocol-defined resuscitation targets and were not treated as independent efficacy endpoints.

Animals were observed for 3 h after completing the relevant shock/resuscitation phase. Those alive at the end of the 180-min observation period were classified as survivors, while earlier deaths were recorded as part of animal disposition. Arterial blood samples were collected according to the assay protocol while the animals were alive. Animals that survived to the scheduled terminal sampling were euthanized under deep pentobarbital anesthesia with an intraperitoneal overdose of 1% pentobarbital sodium (3 mL/100 g), and ileal tissue approximately 5 cm proximal to the cecum was collected. Death was confirmed by the absence of corneal and limb-withdrawal reflexes and persistent apnea for at least 3 min before dissection.

### 2.4 Superior mesenteric artery ischemia-reperfusion control model

In SEC animals, the superior mesenteric artery was exposed through a midline laparotomy and occluded with an atraumatic vascular clamp for 15, 30, or 45 min according to subgroup assignment The clamp was then released to initiate reperfusion. Surgical deaths were recorded separately from post-procedure deaths. Animals that completed surgery entered the observation and assay-specific sampling protocol. Tissue was processed as in the shock groups.

### 2.5 Exploratory bioinformatic analysis

Public ischemia-reperfusion datasets GSE37013, GSE190581, and GSE163638 were obtained from the Gene Expression Omnibus. For GSE37013, expression data were preprocessed with log2 transformation where required and quantile normalization. Principal component analysis (PCA) was performed on the 3,000 features with the highest variance across samples using the R function prcomp with feature scaling enabled; the percentages of total variance explained by PC1 and PC2 are shown on the figure axes. Group centroids, sample-to-centroid segments, and 68% data ellipses were added for visualization. Temporal expression patterns across control, ischemia, 30-min reperfusion (R30), and 120-min reperfusion (R120) were summarized using row-scaled expression and k-means clustering (*k* = 4). Differential expression between ischemia and control used thresholds of adjusted *P* < 0.05 and absolute log2 fold change ≥0.5. These GSE37013 analyses are presented in Figure 2.

**Figure 2:**
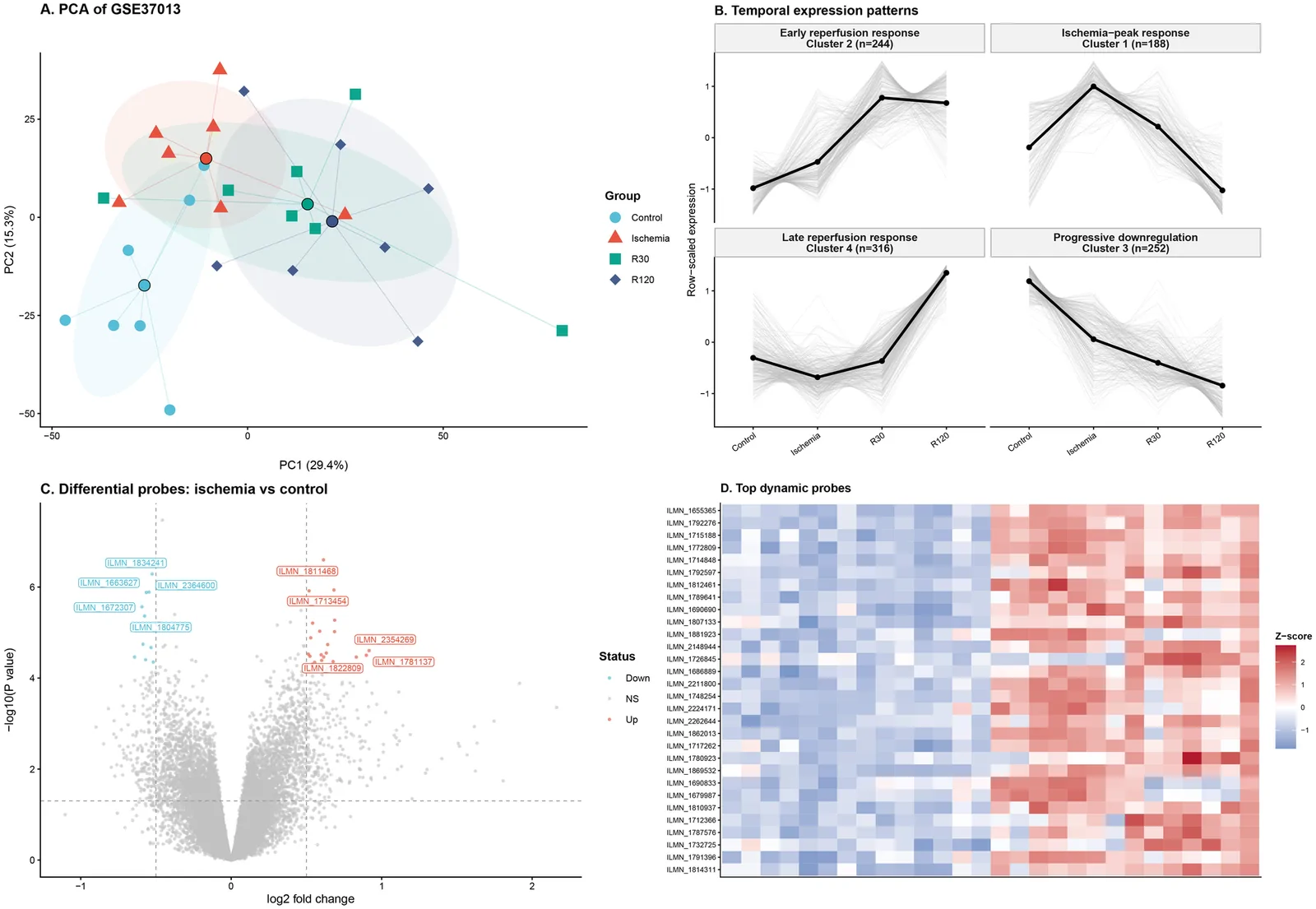
Transcriptomic remodeling during intestinal ischemia-reperfusion in GSE37013. **(A)** PCA of Control, Ischemia, R30, and R120 samples using the 3,000 features with the highest across-sample variance after preprocessing and quantile normalization. Percent variance explained by PC1 and PC2 is shown on the axes; points represent samples, larger points represent group centroids, and shaded regions indicate 68% data ellipses. **(B)** Temporal clustering of row-scaled expression features across Control, Ischemia, R30, and R120; gray lines represent individual features and black lines represent cluster centroids. **(C)** Volcano plot of differential expression between Ischemia and Control at adjusted *P* < 0.05 and absolute log2 fold change ≥0.5. **(D)** Heatmap of top dynamic probes across Control, Ischemia, R30, and R120; colors indicate row-scaled expression (Z-scores).

Single-cell RNA-seq datasets GSE190581 and GSE163638 were analyzed in Seurat v5.0 using quality control, SCTransform normalization, PCA/UMAP dimensionality reduction, and marker-based cell annotation. Broad intestinal cell populations were defined with canonical markers including Epcam/Krt8 (epithelial), Pecam1/Cdh5 (endothelial), Col1a1/Dcn (stromal), and Ptprc/Lyz2 (immune). Module scores were calculated for hypoxia-, apoptosis-, autophagy/mitophagy-, and barrier-repair-associated gene sets. These public-data analyses were exploratory; the single-cell analyses shown in Figure 3 were used to establish cell-type context and biological plausibility, not regulatory causality.

**Figure 3:**
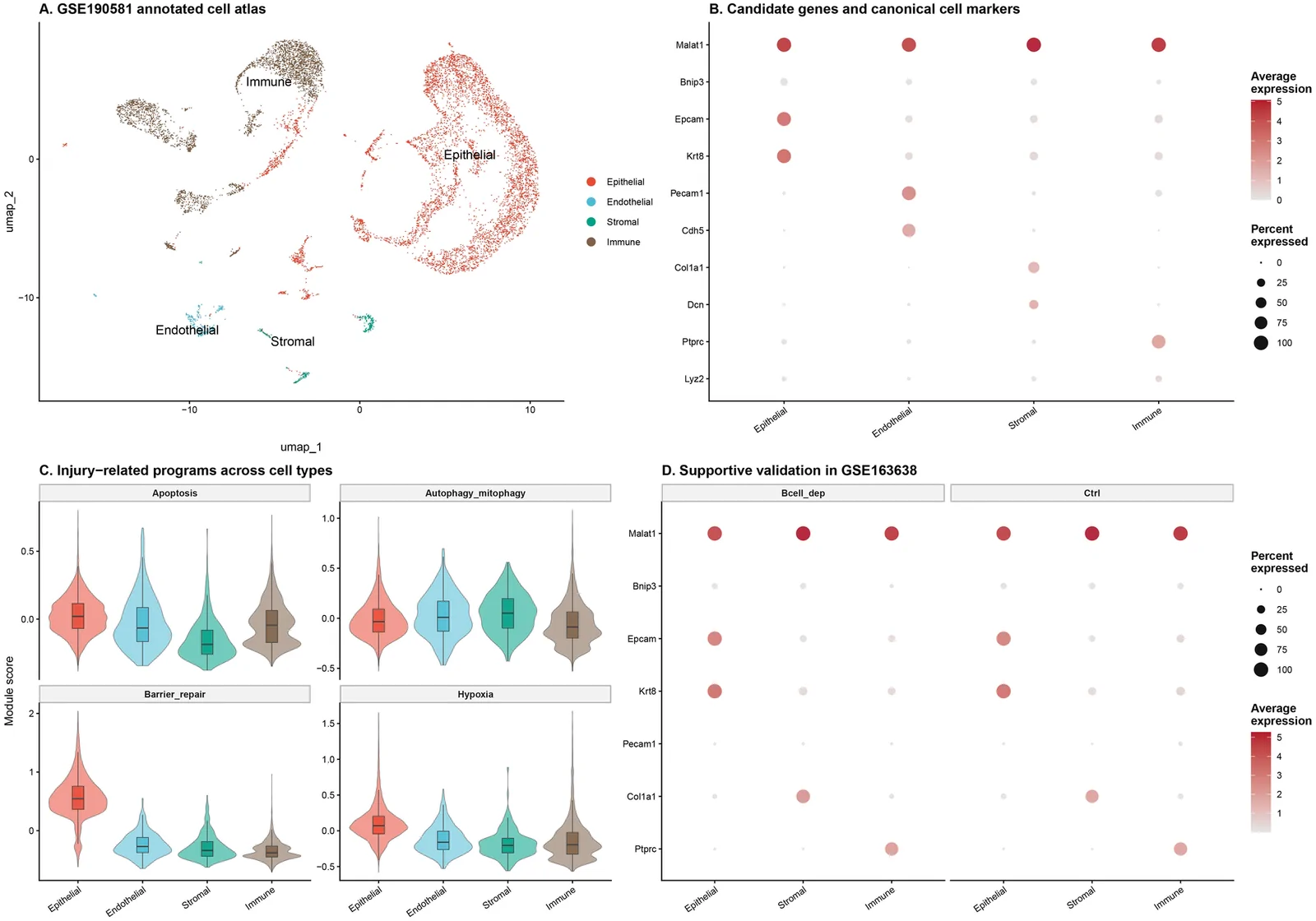
Single-cell localization of Malat1, Bnip3, and injury-related transcriptional programs in intestinal cell populations. **(A)** UMAP representation of GSE190581 after quality control, annotated into epithelial, endothelial, stromal, immune, and other/unknown populations using canonical markers. **(B)** Dot plot showing Malat1, Bnip3, and representative lineage markers; dot size represents the percentage of cells expressing each gene and color intensity represents the average normalized expression. **(C)** Cell-level module-score distributions for hypoxia, apoptosis, autophagy/mitophagy, and barrier-repair programs. **(D)** Supportive analysis of Malat1, Bnip3, and lineage-marker expression in GSE163638. These analyses provide cell-type context and biological plausibility, not causal inference.

### 2.6 Quantitative real-time PCR

Total RNA was extracted from rat ileal mucosa using TRIzol reagent (Invitrogen, Cat. No. 15596026CN). RNA concentration and purity were assessed with a NanoDrop spectrophotometer. For MALAT1 and Bnip3, cDNA was synthesized using a reverse-transcription kit (Thermo Scientific, Cat. No. K1691) with β-actin as the endogenous control. For miR-145-5p, cDNA was generated using a stem-loop primer and miRNA reverse-transcription kit (Thermo Scientific, Cat. No. 4366596) with U6 snRNA as the endogenous control. Quantitative PCR was performed using SYBR Green qPCR Master Mix (Thermo Scientific, Cat. No. K0251). Primer sequences and reference accession numbers are provided in Supplementary Table S1.

Three technical Ct measurements were obtained for the reference and target transcripts in each biological sample and averaged within each animal. *Δ*Ct was calculated as Ct(target)-Ct(reference). Relative expression was determined using the 2^−*ΔΔ*Ct^ method and rescaled for graphical presentation so that the NC group mean equaled 1. qRT-PCR included *n* = 3 independent biological samples per group.

### 2.7 Western blotting

Ileal tissue was homogenized in RIPA buffer (Thermo Scientific, Cat. No. 89900) with added protease inhibitors. Protein concentration was measured using a BCA assay. Equal amounts of protein (20−40 μg per lane) were separated by SDS-PAGE and transferred to PVDF membranes. Membranes were blocked in 5% non-fat milk in TBST for 1 h and incubated overnight at 4 °C with primary antibodies against BNIP3 (Abcam, ab109362; 1:1,000) and β-actin (Abcam, ab8227; 1:5,000), followed by HRP-conjugated secondary antibodies. Signals were detected by enhanced chemiluminescence. Densitometry was performed using ImageJ, and BNIP3 band intensity was normalized to *β*-actin. Three independent biological samples per group were analyzed.

### 2.8 ELISA

Serum IL-6 and TNF-α levels were measured using commercial ELISA kits (COIBO, China; Cat. No. CB11103-Ra and ECH009) according to the manufacturers’ instructions. Absorbance was read at 450 nm, and concentrations were calculated from standard curves. The ELISA included eight independent biological samples per group.

### 2.9 Histopathology and representative semi-quantitative assessment

Ileal segments were fixed in 4% paraformaldehyde for 24 h, dehydrated through graded ethanol, cleared in xylene, paraffin-embedded, sectioned at 4 μm, and stained with hematoxylin and eosin. Sections were examined at 100× and 200× magnification. Representative images from the NC, HS, CFR, RFR, and SEC groups were selected to illustrate the characteristic histopathological features.

For semi-quantitative histopathological assessment, 200× H&E images from three independent animals per group were evaluated. Four domains were scored for each animal: villus epithelial injury, inflammatory cell infiltration, tissue edema, and vascular congestion/hemorrhage. Each component was scored from 0 to 4, yielding a total histopathological injury score ranging from 0 to 16, with higher scores indicating more severe injury. The resulting animal-level scores were used for the analyses described in Section 3.4. The source H&E images used for scoring are provided in the Supplementary Materials.

**Figure 5:**
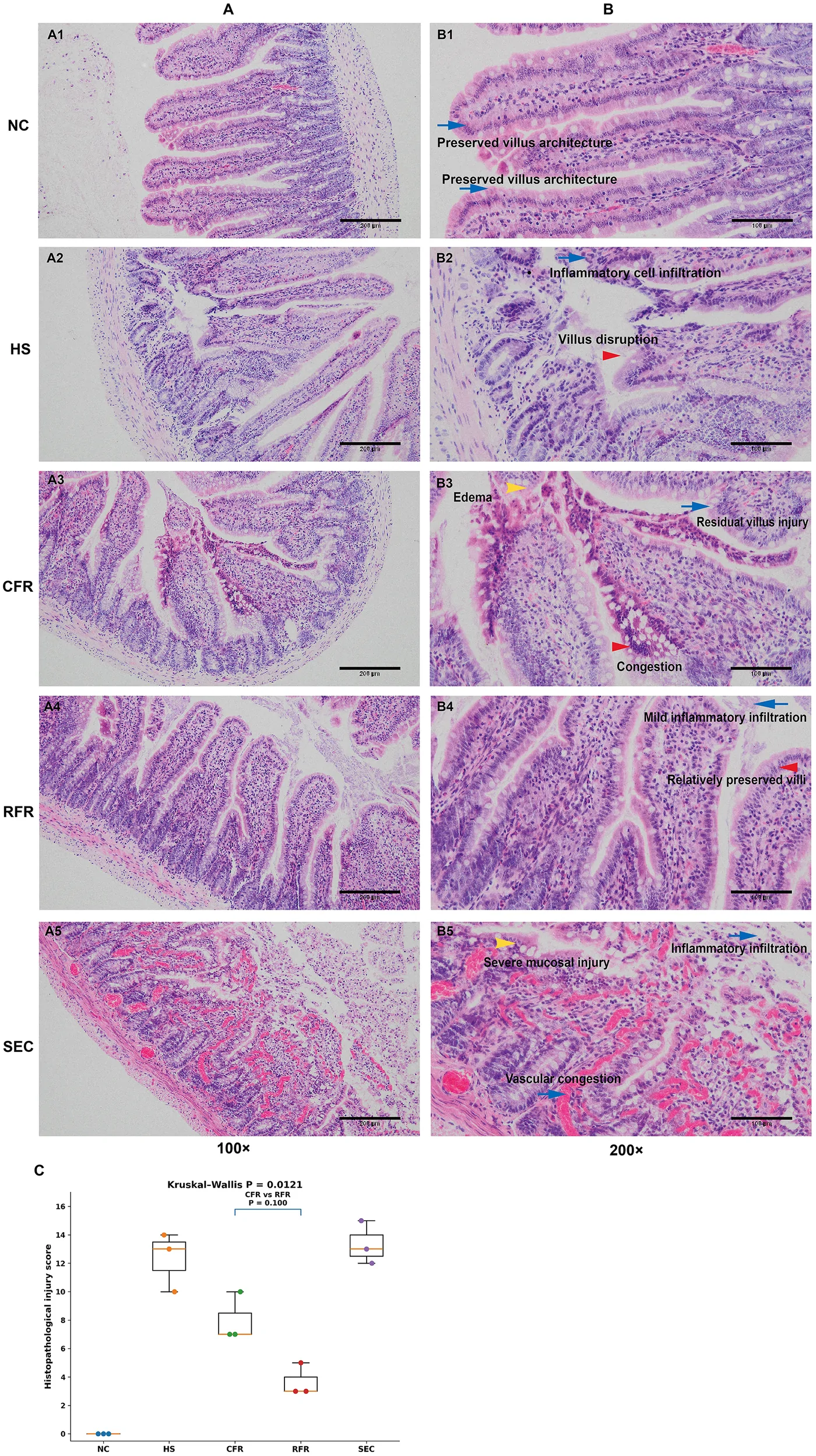
Representative histopathological changes and semi-quantitative injury scores in ileal sections. **(A,B)** Representative hematoxylin and eosin (H&E)-stained ileal sections from the NC, HS, CFR, RFR, and SEC groups are shown at 100× **(A)** and 200× **(B)** magnification. For each group, the 100× and 200× images were obtained from the same representative ileal specimen at different magnifications. The 200× images are annotated to indicate characteristic histopathological features, including villus disruption/injury, inflammatory cell infiltration, edema, and vascular congestion, as appropriate. NC showed preserved villus architecture; HS and SEC showed more severe mucosal injury; CFR showed residual villus injury, edema, and congestion; and RFR showed relatively preserved villus architecture with milder histological abnormalities. Scale bars: 200 μm at 100× and 100 μm at 200×. **(C)** Semi-quantitative histopathological injury scores from three independent animals per group. Villus epithelial injury, inflammatory infiltration, edema, and vascular congestion/hemorrhage were each scored from 0 to 4, yielding a total score of 0–16 per animal. Individual animal values are shown with box plots. Overall group differences were assessed using the Kruskal–Wallis test (*P* = 0.0121); the prespecified CFR-versus-RFR comparison used a two-sided exact Mann–Whitney *U* test (*P* = 0.100).

### 2.10 TUNEL staining and representative image-based analysis

Apoptotic DNA fragmentation was visualized using a TUNEL kit (Roche, Germany) according to the manufacturer's protocol. After deparaffinization and antigen retrieval in sodium citrate buffer (pH 6.0), sections were blocked with 3% H_2_O_2_ for 10 min and incubated with TUNEL reaction mixture at 37°C for 60 min. FITC-labeled dUTP was used for fluorescent detection with DAPI counterstaining. Images were captured at 400× magnification.

For the supplemental descriptive analysis, representative merged images underwent image-level nuclear segmentation. DAPI-positive nuclei were detected, and nuclear TUNEL-associated green fluorescence was classified using a common threshold derived from the NC background distribution. The image-level apoptotic index was calculated as TUNEL-positive nuclei divided by detected DAPI-positive nuclei×100%. Because the analyzed images were representative fields rather than a complete animal-level replicate series, no inferential statistics were applied. Results are shown in Supplementary Figure S2.

### 2.11 Statistical analysis

The individual animal served as the biological unit of analysis. Technical replicates and repeated assay measurements from the same animal were not treated as independent observations. Continuous data are presented as mean ± SD, with individual biological data points where applicable. Statistical methods were specified based on the type and scale of each outcome, rather than selected solely on the basis of formal normality testing. The primary treatment comparison was RFR versus CFR; comparisons involving NC, HS, and SEC were contextual or exploratory unless stated otherwise.

Three-hour survival was analyzed as a binary animal-level outcome using a two-sided Fisher exact test for the prespecified CFR-versus-RFR comparison, with the odds ratio and 95% confidence interval reported. A combined Kaplan–Meier analysis was not performed because exact individual death times were unavailable for all animals across both batches.

Coagulation variables (PT, TT, APTT, INR, and PA) were analyzed using the Kruskal–Wallis test for overall five-group differences, followed by Dunn pairwise comparisons with Holm adjustment across the 10 comparisons within each endpoint. Coagulation data are presented as median (interquartile range).

For serum IL-6 and TNF-α, overall, five-group comparisons used Welch one-way ANOVA with Games-Howell *post hoc* testing. The prespecified CFR-versus-RFR contrast was assessed using a two-sided Welch *t*-test, with mean differences and 95% confidence intervals.

For qRT-PCR, inference was performed on animal-level *Δ*Ct values, not on transformed fold-change values. Exploratory overall five-group comparisons used Welch one-way ANOVA; the prespecified CFR-versus-RFR contrast used a two-sided Welch *t*-test. Western blot densitometry was analyzed in the same way using normalized BNIP3/β-actin ratios. Because qRT-PCR and Western blotting included *n* = 3 biological samples per group, these analyses are considered exploratory.

For the H&E histopathological injury score, three independent animals per group were analyzed. Overall differences among the five groups were assessed using the Kruskal–Wallis test, and the prespecified CFR-versus-RFR comparison was assessed using a two-sided exact Mann–Whitney *U* test. The TUNEL semi-quantitative analysis remained a descriptive representative-image analysis and was not tested inferentially. All tests were two-sided. Exact *P* values are reported where practical, with *P* < 0.05 considered statistically significant. No outliers were excluded, and no missing data were imputed unless a measurement had been prospectively documented as a technical assay failure. Analyses were performed in R v4.6.3 and SPSS v26.0.

## 3 Results

### 3.1 Animal disposition and 3-h survival

The 100 animals were studied in two batches, and no additional animals were introduced as replacements. Across both batches, 3-h survival was 100% (10/10) in NC, 40% (8/20) in HS, 60% (12/20) in CFR, and 75% (15/20) in RFR. In the prespecified CFR-versus-RFR comparison, the difference was not statistically significant (two-sided Fisher exact test, *P* = 0.501; odds ratio, 2.00; 95% CI, 0.52–7.72). The numerically higher survival in RFR is therefore descriptive and does not indicate a survival benefit.

In the SEC group, eight of the 30 initially allocated animals died during surgery. Among the 22 that completed surgery, 18 survived the subsequent 3-h observation period (81.8%). Batch-specific animal disposition and assay allocation are summarized in Supplementary Figure S3.

### 3.2 Coagulation profiles differed between the two resuscitation strategies

Coagulation measurements were available for all 36 animals in the dedicated coagulation dataset (NC, *n* = 4; HS, *n* = 8; CFR, *n* = 8; RFR, *n* = 8; SEC, *n* = 8). Overall group differences were significant for PT (Kruskal–Wallis *H* = 17.70, *P* = 0.0014), TT (*H* = 21.67, *P* = 0.00023), APTT (*H* = 26.80, *P* < 0.0001), INR (*H* = 21.23, *P* = 0.00029), and PA (*H* = 20.70, *P* = 0.00036) (Supplementary Table S2).

In the prespecified CFR-versus-RFR comparison, PT was shorter in RFR [median (IQR), 14.70 (13.80–15.88) vs. 23.45 (21.80–27.38) s; Dunn–Holm adjusted *P* = 0.034], as was TT [19.35 (18.25–19.70) vs. 29.50 (24.78–33.65) s; adjusted *P* = 0.008]. APTT was numerically shorter in RFR [38.05 (36.45–39.62) vs. 55.45 (39.60–75.53) s], but the difference did not survive multiplicity adjustment (adjusted *P* = 0.422). Differences in INR (adjusted *P* = 0.395) and PA (adjusted *P* = 0.289) were also non-significant after Holm correction.

### 3.3 Lower systemic inflammatory cytokines after restrictive resuscitation

Serum IL-6 and TNF-α differed markedly among groups (*n* = 8 independent biological samples per group; Figures 4C,D). Welch one-way ANOVA showed significant overall group effects for IL-6 (*F* = 726.29, *P* < 0.0001) and TNF-α (*F* = 392.69, *P* < 0.0001). In the prespecified CFR-versus-RFR comparison, IL-6 was lower in RFR (89.71 ± 4.49 vs. 115.41 ± 4.07 pg/mL; mean difference, −25.71 pg/mL; 95% CI, −30.30 to −21.11; Welch *t*-test, *P* < 0.0001), as was TNF-α (125.81 ± 5.88 vs. 152.04 ± 6.47 pg/mL; mean difference, −26.22 pg/mL; 95% CI, −32.86 to −19.58; *P* < 0.0001). Games-Howell *post hoc* analysis confirmed the same direction of effect after accounting for multiple group comparisons.

**Figure 4:**
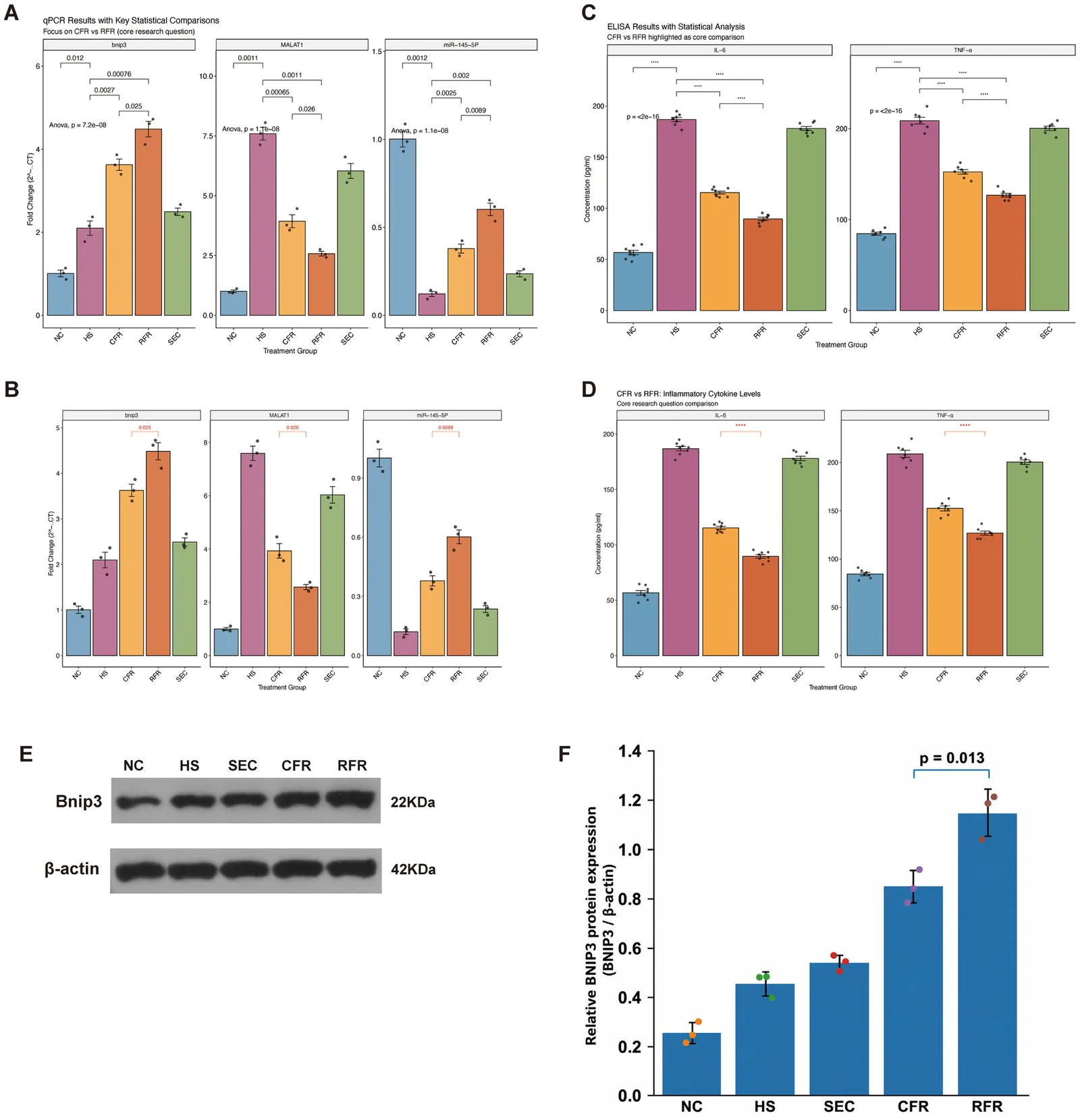
Molecular, inflammatory, and BNIP3 protein profiles across experimental groups. **(A,B)** Relative expression of MALAT1, miR-145-5p, and Bnip3 in ileal tissue by qRT-PCR (*n* = 3 independent biological replicates per group). Three technical Ct replicates were averaged within each animal. MALAT1 and Bnip3 were normalized to β-actin, and miR-145-5p to U6 snRNA. Comparisons were performed on animal-level *Δ*Ct values; 2^−*ΔΔ*Ct^ values are shown graphically with the NC mean normalized to 1. In the prespecified CFR-versus-RFR comparison, MALAT1 was lower in RFR (*P* = 0.011) while miR-145-5p (*P* = 0.005) and Bnip3 (*P* = 0.021) were higher. **(C,D)** Serum IL-6 and TNF-α by ELISA (*n* = 8 independent biological replicates per group). Overall group differences were assessed by Welch one-way ANOVA with Games-Howell *post hoc* testing; CFR-versus-RFR comparisons used two-sided Welch *t*-tests (*P* < 0.0001 for both cytokines). **(E,F)** Representative BNIP3 Western blot and densitometric quantification (*n* = 3 independent biological replicates per group). BNIP3 band intensity was normalized to *β*-actin; bars represent mean ± SD and individual dots represent biological replicates. The prespecified CFR-versus-RFR comparison used a two-sided Welch *t*-test (*P* = 0.013). Molecular analyses with *n* = 3 per group were exploratory.

### 3.4 Representative ileal sections showed differential patterns of mucosal injury

H&E staining demonstrated preserved villus architecture and organized epithelium in NC (Figures 5A,B). HS sections exhibited prominent villus disruption, epithelial disorganization, inflammatory cell infiltration, and mucosal edema. CFR sections showed residual villus injury, edema, congestion, and inflammatory infiltration, whereas the representative RFR sections showed relatively better-preserved villus architecture with milder histopathological abnormalities. SEC sections exhibited severe ischemia-reperfusion-associated mucosal injury with prominent vascular congestion and inflammatory infiltration. Representative lesions are annotated in the 200× images in Figure 5B.

Semi-quantitative analysis of H&E sections from three independent animals per group revealed significant overall differences in histopathological injury scores among the five groups (Kruskal–Wallis H = 12.84, *P* = 0.0121; Figure 5C). Median injury scores were 0 in NC, 13 in HS, seven in CFR, three in RFR, and 13 in SEC. In the prespecified CFR-versus-RFR comparison, injury scores were numerically lower in RFR, although the difference did not reach statistical significance (exact Mann–Whitney *U* test, *P* = 0.100).

### 3.5 TUNEL positivity in representative sections

Representative TUNEL images showed minimal labeling in NC and more extensive TUNEL-associated fluorescence after hemorrhagic shock (Figure 6). In the representative CFR and RFR fields, TUNEL-associated fluorescence appeared more extensive in CFR and less extensive and more scattered in RFR. SEC showed substantial labeling consistent with ischemia-reperfusion injury. In the merged-image analysis, image-level apoptotic indices were 5.2% in NC, 43.9% in HS, 17.6% in CFR, 11.9% in RFR, and 23.5% in SEC (Supplementary Figure S2). These are descriptive image-level estimates and were not used for animal-level inferential testing.

### 3.6 MALAT1, miR-145-5p, and BNIP3 changed in a coordinated pattern

qRT-PCR showed coordinated changes in MALAT1, miR-145-5p, and Bnip3 expression across groups (*n* = 3 biological replicates per group; Figures 4A,B). Comparisons were performed on animal-level *Δ*Ct values, while relative expression is displayed after 2^−*ΔΔ*Ct^ normalization with the NC group mean set to 1. In the prespecified CFR-versus-RFR comparison, MALAT1 was lower in RFR (2.57 ± 0.17 vs. 3.92 ± 0.47; Welch *t*-test on *Δ*Ct, *P* = 0.011), whereas miR-145-5p (0.60 ± 0.06 vs. 0.38 ± 0.04; *P* = 0.005) and Bnip3 mRNA (4.46 ± 0.32 vs. 3.60 ± 0.23; *P* = 0.021) were higher. An exploratory five-group Welch ANOVA identified overall group differences for all three measurements (all *P* < 0.001).

**Figure 6:**
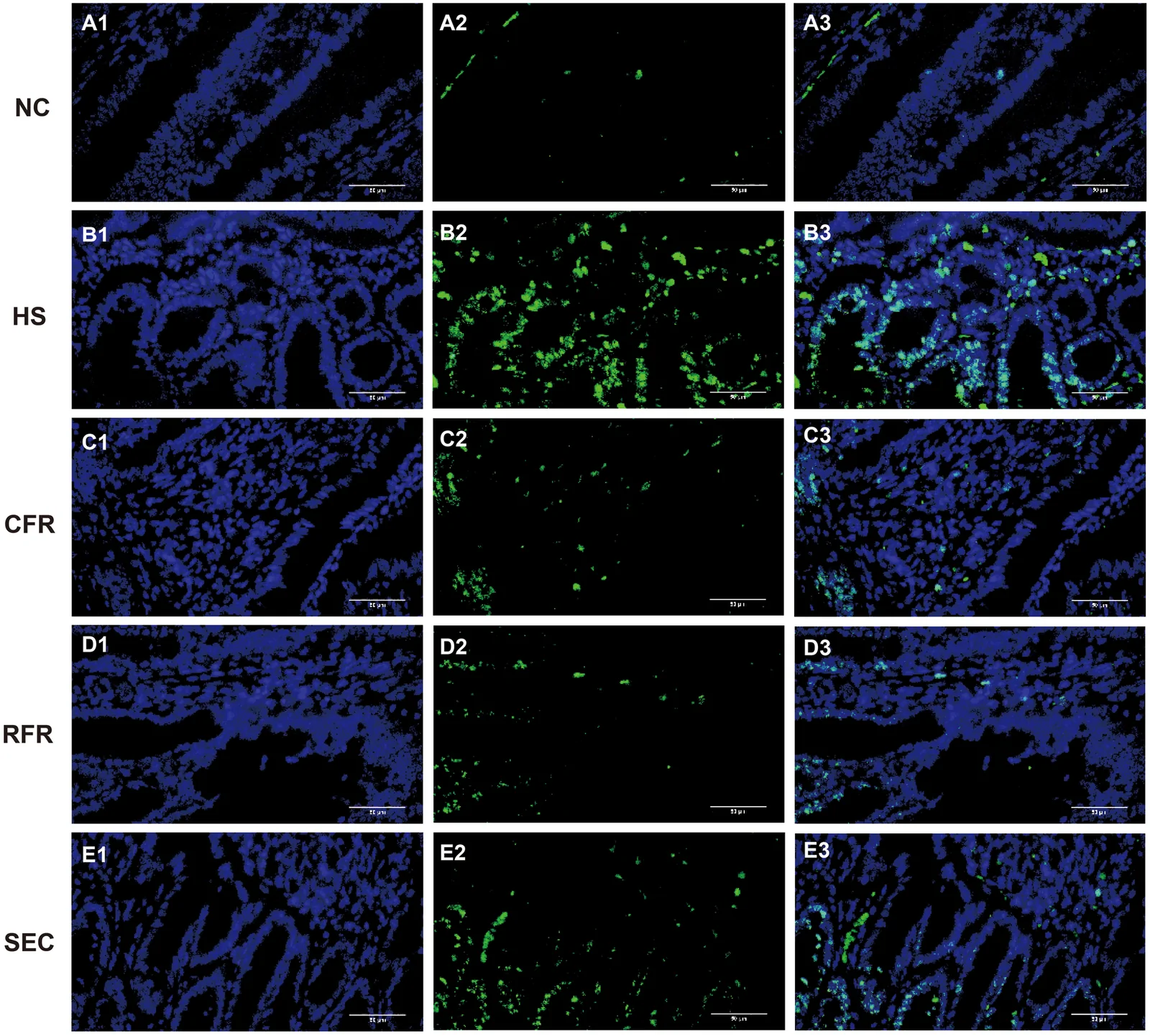
Representative TUNEL staining of ileal sections across experimental groups. Representative fluorescence images of ileal sections from the NC, HS, CFR, RFR, and SEC groups are shown at 400× magnification. For each group, the three panels in the same row show matched fluorescence channels acquired from the same representative specimen and the same microscopic field, including DAPI nuclear staining (blue), TUNEL-associated fluorescence (green), and the corresponding merged image. Representative images showed relatively few TUNEL-positive signals in the NC group, more prominent TUNEL-associated fluorescence in the HS group, and visually fewer positive signals in the resuscitation groups. These representative image-based observations are descriptive and should not be interpreted as animal-level inferential comparisons. Scale bar: 50 μm. Descriptive image-based semi-quantitative analysis is presented in Supplementary Figure S2.

Western blotting confirmed the Bnip3 mRNA data (Figure 4E). Densitometry normalized to β-actin revealed BNIP3 protein levels of 0.237 ± 0.042 in NC, 0.439 ± 0.049 in HS, 0.842 ± 0.066 in CFR, 1.144 ± 0.093 in RFR, and 0.525 ± 0.032 in SEC. In the prespecified CFR-versus-RFR comparison, BNIP3 was higher in RFR (mean difference, 0.302; 95% CI, 0.112-0.492; Welch *t*-test, *P* = 0.013).

miR-145-5p and BNIP3 thus increased in parallel in RFR. This does not align with the directional prediction of a simple linear MALAT1-miR-145-5p-BNIP3 ceRNA model, and we interpret it as coordinated expression rather than a validated regulatory axis.

### 3.7 Public transcriptomic datasets place the candidate molecules in cellular context

Analysis of GSE37013 showed broad transcriptomic remodeling during intestinal ischemia-reperfusion (Figure 2). PCA separated control, ischemia, R30, and R120 samples, with variance explained by PC1 and PC2 shown on the figure axes. Time-resolved clustering identified ischemia-peak, early-reperfusion, late-reperfusion, and progressively downregulated expression patterns, and differential-expression analysis confirmed widespread remodeling between ischemic and control samples.

Single-cell analyses then addressed the cellular context of the candidate molecules (Figure 3). In GSE190581, epithelial, endothelial, stromal, and immune populations were identified; Malat1 was broadly detected across major cell types, and Bnip3 was expressed in both epithelial and non-epithelial compartments. Hypoxia-, apoptosis-, autophagy/mitophagy-, and barrier-related transcriptional programs showed cell-type-specific distributions. The broad expression of Malat1 and Bnip3 was also observed in GSE163638. These analyses support biological plausibility, but do not establish a direct regulatory relationship.

## 4 Discussion

Restrictive resuscitation with 7.5% hypertonic saline was associated with a more favorable acute intestinal injury phenotype than conventional isotonic resuscitation. Representative histological sections suggested better preservation of villus architecture with less evident edema and inflammatory infiltration, while representative TUNEL fields showed lower apparent positivity. Circulating IL-6 and TNF-α were also lower. Three-hour survival was higher under RFR (75% vs. 60%), but the study was not powered for mortality, and the difference was not significant. The coagulation effect was partial: PT and TT were shorter under RFR, whereas APTT, INR, and PA did not differ between groups. At the molecular level, RFR was associated with lower MALAT1 and higher miR-145-5p and Bnip3/BNIP3 expression—a coordinated shift that contradicts a simple linear MALAT1-miR-145-5p-BNIP3 ceRNA model. The exploratory analyses of public transcriptomic data place these molecules in a cellular context without addressing causality.

### 4.1 Survival and coagulation

Three-hour survival favored RFR numerically (75% vs. 60%), but the difference was not statistically significant, and the confidence interval around the odds ratio was wide. Survival was recorded as an animal-disposition outcome, not as a powered efficacy endpoint, and should be interpreted in the physiological context of the tissue findings.

The coagulation data indicate two distinct acute laboratory profiles. Overall, five-group differences were observed for PT, TT, APTT, INR, and PA, but in the prespecified CFR-versus-RFR comparison, only PT and TT remained significant after multiplicity adjustment. These data support a distinct coagulation profile under restrictive hypertonic resuscitation, not a general claim that RFR normalizes hemorrhage-associated coagulopathy.

The much smaller crystalloid volume used in RFR is one plausible contributor. Large-volume isotonic crystalloid dilutes coagulation factors and worsens coagulation abnormalities, while restrictive resuscitation limits that exposure (5, 6, 16, 17). Even so, this experiment cannot separate fluid volume, hypertonicity, and the lower MAP target as explanations for the PT/TT differences. Systemic heparin and heparinized catheters may also affect absolute coagulation times, particularly APTT and TT. We therefore regard the coagulation results as exploratory laboratory evidence, not as proof that RFR corrects coagulopathy.

### 4.2 The intestinal injury phenotype

The inflammatory findings were directionally consistent with the descriptive morphological observations. Compared to CFR, RFR showed lower circulating IL-6 and TNF-α, while representative histological sections suggested better preservation of villus architecture with less evident edema and inflammatory infiltration, and representative TUNEL fields showed lower apparent positivity.

This is biologically plausible because intestinal injury after hemorrhagic shock depends on more than the restoration of systemic arterial pressure. Ischemia causes epithelial energetic failure and barrier disruption, while reperfusion amplifies oxidative stress, inflammatory activation, endothelial dysfunction, and edema (1–4). Conventional isotonic crystalloid quickly restores macrocirculatory pressure but adds hydrostatic load and interstitial edema. Hypertonic saline achieves intravascular expansion with a much smaller infused volume and has been reported to exert immunomodulatory effects. Both properties are relevant to mucosal microcirculation and epithelial oxygen delivery.

The present design cannot identify which component of RFR produced this phenotype because RFR differed from CFR in infused volume, tonicity, and target MAP simultaneously. Volume-matched isotonic controls, tonicity-matched controls, and pressure-matched treatment arms would be needed to separate them. For now, we can claim that the combined restrictive hypertonic strategy was associated with less severe intestinal injury under these conditions.

### 4.3 Interpreting the MALAT1, miR-145-5p, and BNIP3 pattern

The molecular data add a second layer to the phenotype. Previous work supports examining these three molecules together: MALAT1 has been implicated in ischemia-reperfusion responses in several organs and has been reported to interact with miR-145 through competing-endogenous-RNA mechanisms (9–12), and miR-145 has been linked to BNIP3 regulation in other experimental systems (18).

Our data support only part of that framework. Lower MALAT1 with higher miR-145-5p in RFR is directionally consistent with reduced MALAT1-mediated sequestration of miR-145-5p. However, Bnip3 mRNA and BNIP3 protein also increased. If miR-145-5p-mediated repression were the dominant determinant of BNIP3 expression here, then higher miR-145-5p should have led to a decrease in BNIP3. The observed parallel increase suggests that these data so not support a simple linear ceRNA axis.

Several explanations for the discordance are possible. A MALAT1-miR-145-5p interaction may exist without being the dominant regulator of BNIP3 during acute hemorrhage and reperfusion. BNIP3 is strongly responsive to hypoxic and mitochondrial stress signals, which may outweigh miRNA-mediated repression in an acute observation window. Additionally, bulk-tissue qRT-PCR and Western blotting average signals across epithelial, endothelial, stromal, and immune compartments, whose molecular dynamics may differ.

We therefore describe the three molecules as coordinately altered candidate stress-response molecules. Demonstrating direct interaction will require functional perturbation: MALAT1 knockdown or overexpression, miR-145-5p mimic and inhibitor experiments, and reporter assays targeting the rat Bnip3 3′ UTR.

### 4.4 Higher BNIP3 alongside lower TUNEL positivity

BNIP3 protein levels were higher in RFR, whereas representative TUNEL fields showed lower apparent positivity, and representative histological sections suggested better preservation of mucosal architecture. BNIP3 is often described as a pro-death BCL2-family protein, but it also participates in mitochondrial quality control and mitophagy (14, 15, 19). We hypothesize that this increase represents a compensatory enhancement of mitophagy: Clearing depolarized mitochondria during early reperfusion would limit secondary reactive oxygen species generation and the pro-death signaling it triggers.

We cannot demonstrate this mechanism here. LC3-II/I, p62/SQSTM1 turnover, mitochondrial membrane potential, mitophagic structures on electron microscopy, and cleaved caspase-3 were all unmeasured. TUNEL, moreover, detects DNA fragmentation only in nuclei retained within the section and misses cells lost to severe shedding or necrosis, so lower TUNEL positivity does not reflect cell-death flux. Based on the present evidence, BNIP3 upregulation accompanies the RFR-associated stress response and cannot be classified as either protective or pro-apoptotic (20).

### 4.5 Scope of the public transcriptomic analyses

The public datasets were used deliberately as contextual rather than confirmatory evidence. GSE37013 demonstrated broad transcriptional remodeling during intestinal ischemia-reperfusion, and the single-cell datasets showed that Malat1 and Bnip3 are expressed in the intestinal cell populations relevant to the *in vivo* phenotype. Injury-associated programs involving hypoxia, apoptosis, autophagy/mitophagy, and barrier regulation were distributed across the major cell compartments (21).

Together, this supports MALAT1 and BNIP3 as components of the intestinal ischemia-reperfusion response and suggests why bulk-tissue measurements need not follow a simple regulatory model. The datasets nonetheless differ from our experiment in species, injury context, platform, and sampling design. They validate neither the RFR intervention nor a direct MALAT1-miR-145-5p-BNIP3 interaction.

### 4.6 Translational implications

These findings relate to acute resuscitation research, but their translational relevance is limited. The intestinal mucosa is highly sensitive to hypoperfusion and reperfusion, and maintaining mucosal integrity may influence systemic inflammation and secondary organ dysfunction. In this rat model, a restricted-volume 7.5% hypertonic saline with a lower MAP target produced a more favorable acute intestinal phenotype than conventional isotonic saline.

However, this does not provide direct evidence for any clinical fluid prescription. Factors such as hypertonic saline concentration, timing, hemorrhage severity, anesthesia, and the absence of concomitant traumatic injuries distinguish this model from clinical trauma, and the study does not separate fluid volume, fluid composition, and pressure target. Its value lies in supporting the broader idea that resuscitation strategy can influence tissue-level injury independently of the extent to which macrocirculatory pressure is restored.

The molecular findings are hypothesis-generating in a similar manner. MALAT1, miR-145-5p, and BNIP3 are candidate indicators of the intestinal stress response, but none has yet been validated as a biomarker or therapeutic target. Larger animal studies with serial sampling, followed by research in human trauma cohorts, will be necessary.

### 4.7 Limitations

Several limitations affect these results. The molecular findings are associative: No functional perturbation of MALAT1, miR-145-5p, or BNIP3 was performed, and the parallel increase in miR-145-5p and BNIP3 argues against a simple linear ceRNA interpretation. qRT-PCR and Western blotting also used small biological sample sizes (*n* = 3 per group), making those analyses are exploratory, and bulk-tissue measurement may obscure cell-specific responses.

The design imposes two additional constraints. RFR and CFR differed simultaneously in fluid volume, tonicity, and MAP target, so the observed differences cannot be attributed to any single component; continuous animal-level MAP trajectories, blood-loss volumes, and resuscitation volumes were not analyzed as independent efficacy endpoints. The observation window was also brief. We recorded 3-h survival and acute tissue and molecular responses, which do not address delayed barrier failure, bacterial translocation, repair, secondary organ dysfunction, or long-term mortality.

Three measurement caveats apply. Systemic heparin and heparinized catheters could influence absolute clotting times, and coagulation was an exploratory laboratory outcome rather than a powered endpoint. The H&E semi-quantitative analysis was based on three independent animals per group and therefore provided animal-level histopathological injury scores, whereas the TUNEL semi-quantitative analysis remained a representative-image analysis and should be interpreted descriptively rather than as a formal animal-level inferential comparison; they describe the visible morphology but are not formal inferential comparisons. Terminal tissue analyses depended on specimen availability — no animals beyond the original 100 were introduced as replacements, but histological and molecular analyses necessarily represent animals from which analyzable specimens could be obtained, while deaths remained in the animal-disposition record.

Finally, the uniform 8-h fast may have affected the basal metabolic or morphological state of the mucosa. The identical protocol across groups makes this an unlikely explanation for the CFR-versus-RFR differences, but a fasting-related contribution to baseline ileal morphology cannot be excluded. The public transcriptomic datasets were heterogeneous in species, injury model, platform, and sampling design and were included to provide context and generate hypotheses, not as an independent validation cohort.

## 5 Conclusion

Under the present experimental conditions, restrictive resuscitation with 7.5% hypertonic saline and a lower MAP target was associated with a more favorable acute intestinal injury phenotype than conventional 0.9% saline resuscitation. This phenotype was supported by lower systemic inflammatory cytokine levels and was descriptively accompanied by better-preserved ileal morphology and lower apparent TUNEL positivity in representative sections and fields. RFR was also associated with lower MALAT1 and higher miR-145-5p and Bnip3/BNIP3 expression. These molecular changes are coordinated but mechanistically unresolved, and the parallel increase in miR-145-5p and BNIP3 does not support a simple linear ceRNA model. Functional and cell-specific studies will be needed to define the regulatory relationships among these molecules and the biological role of BNIP3 after hemorrhagic shock and resuscitation.

## Data Availability

The original contributions presented in the study are included in the article/Supplementary Material, further inquiries can be directed to the corresponding author.

## Supplementary material

Supplementary Figure S1 Source H&E images used for semi-quantitative histopathological injury scoring. H&E-stained ileal sections at 200× magnification from three independent animals in each experimental group (NC, HS, CFR, RFR, and SEC) were used for the animal-level semi-quantitative analysis presented in Figure 5C. Each specimen was evaluated for villus epithelial injury, inflammatory cell infiltration, edema, and vascular congestion/hemorrhage. Scale bar: 100 μm.

Supplementary Figure S2 Representative image-based semi-quantitative analysis of TUNEL positivity in ileal sections. Representative merged TUNEL/DAPI images underwent automated nuclear segmentation. DAPI-positive nuclei were detected, and nuclear TUNEL-associated fluorescence was classified using a common threshold derived from the NC image background distribution. Image-level apoptotic indices were NC 5.2%, HS 43.9%, CFR 17.6%, RFR 11.9%, and SEC 23.5%. These values are descriptive image-level estimates from representative fields rather than independent biological replicate distributions, and no inferential testing was applied.

Supplementary Figure S3 Animal disposition and assay allocation throughout the experimental protocol. A total of 100 rats were allocated to NC (*n* = 10), HS (*n* = 20), CFR (*n* = 20), RFR (*n* = 20), and SEC (*n* = 30) and studied in two batches: Batch 1, *n* = 36 (NC 4, HS 8, CFR 8, RFR 8, SEC 8); Batch 2, *n* = 64 (NC 6, HS 12, CFR 12, RFR 12, SEC 22). No animals beyond the original 100 were introduced as replacements. Across both batches, 3-h survival was 10/10 (100%) in NC, 8/20 (40%) in HS, 12/20 (60%) in CFR, and 15/20 (75%) in RFR; the CFR-versus-RFR difference was not statistically significant (two-sided Fisher exact test, *P* = 0.501). In SEC, eight of the 30 initially allocated animals died during surgery; among the 22 that completed surgery, 18 survived the subsequent 3-h observation period (81.8%). Assay-specific biological sample sizes are stated in the corresponding Methods sections and figure and table legends.
