# Supplementary material for: Restrictive hypertonic saline resuscitation is associated with less intestinal injury after hemorrhagic shock in rats and with coordinated changes in MALAT1, miR-145-5p, and BNIP3 expression
Source: Front Surg. 2026 Sep 30;13:1920366. doi: 10.3389/fsurg.2026.1920366 (PMC13662056; doi:10.3389/fsurg.2026.1920366)
Supplement: Supplementary file 1 [file Datasheet1.pdf]

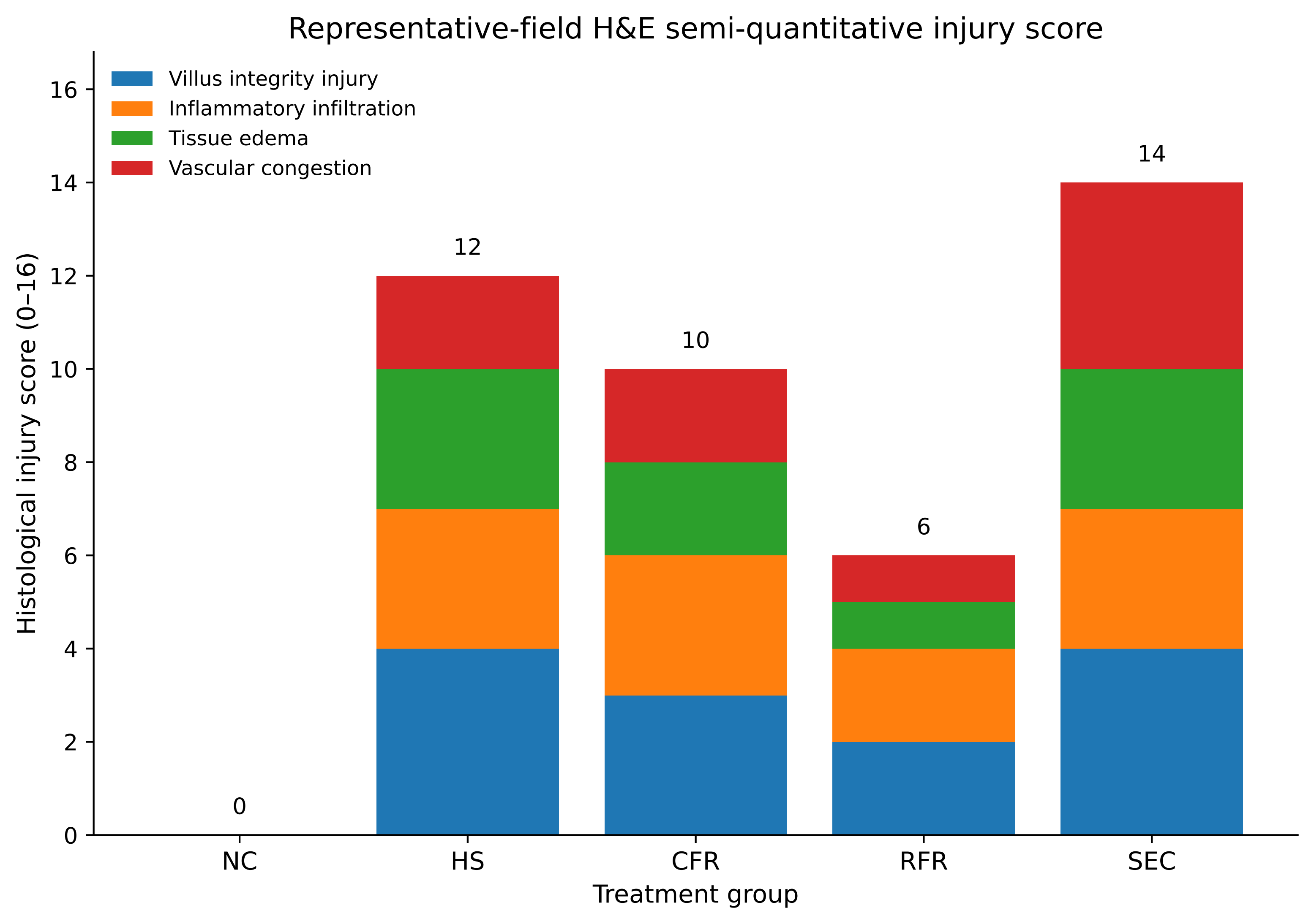

Representative-field H&E semi-quantitative injury score
Villus integrity injury
Inflammatory infiltration
Tissue edema
Vascular congestion
Histological injury score (0–16)
0
2
4
6
8
10
12
14
16
0
12
10
6
14
NC
HS
CFR
RFR
SEC
Treatment group
