# Supplementary material for: Restrictive hypertonic saline resuscitation is associated with less intestinal injury after hemorrhagic shock in rats and with coordinated changes in MALAT1, miR-145-5p, and BNIP3 expression
Source: Front Surg. 2026 Sep 30;13:1920366. doi: 10.3389/fsurg.2026.1920366 (PMC13662056; doi:10.3389/fsurg.2026.1920366)
Supplement: Supplementary file 2 [file Datasheet2.pdf]

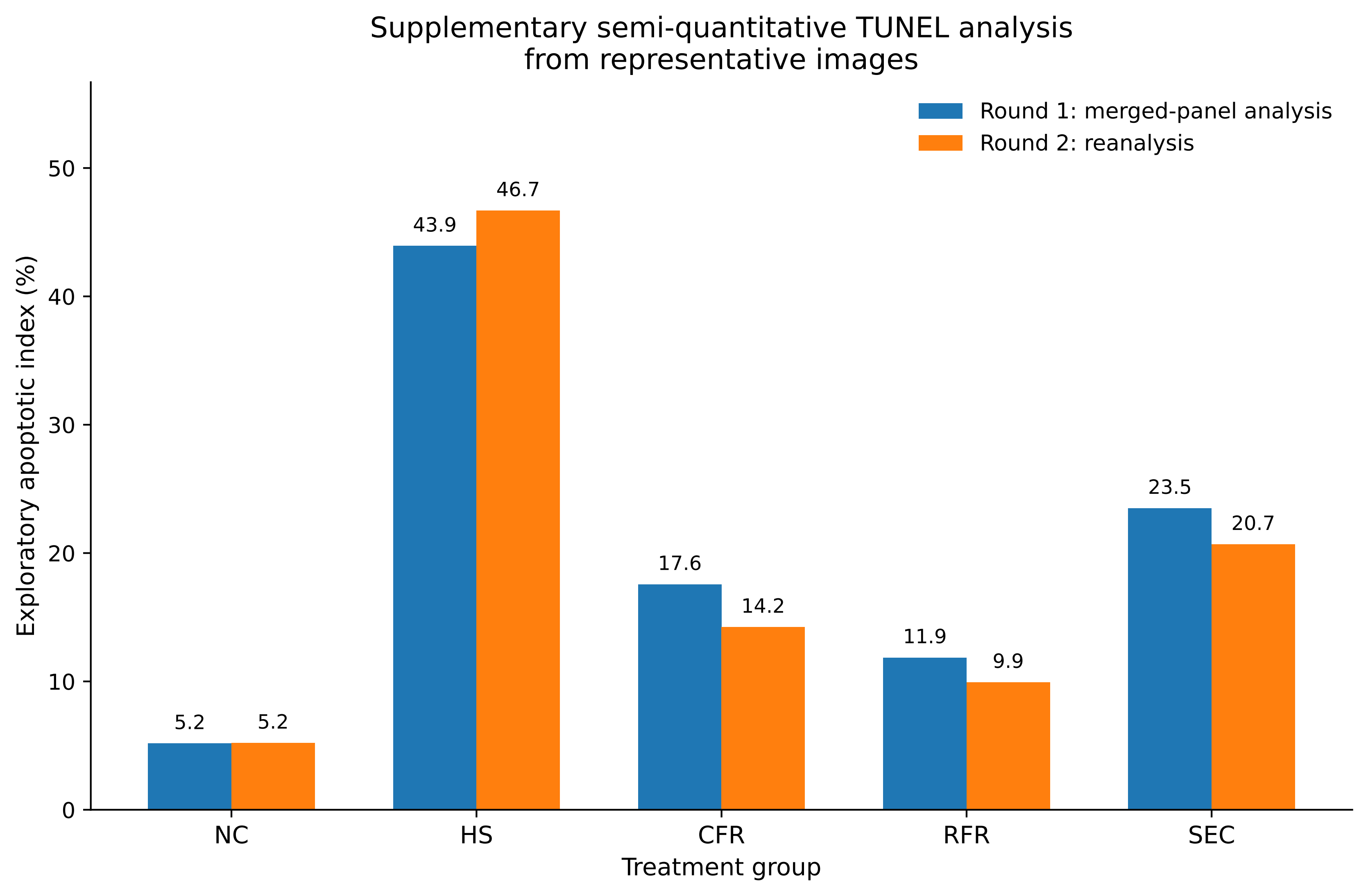

Supplementary semi-quantitative TUNEL analysis
from representative images
Round 1: merged-panel analysis
Round 2: reanalysis
Exploratory apoptotic index (%)
0
10
20
30
40
50
5.2
5.2
43.9
46.7
17.6
14.2
11.9
9.9
23.5
20.7
NC
HS
CFR
RFR
SEC
Treatment group
