# Supplementary material for: Restrictive hypertonic saline resuscitation is associated with less intestinal injury after hemorrhagic shock in rats and with coordinated changes in MALAT1, miR-145-5p, and BNIP3 expression
Source: Front Surg. 2026 Sep 30;13:1920366. doi: 10.3389/fsurg.2026.1920366 (PMC13662056; doi:10.3389/fsurg.2026.1920366)
Supplement: Supplementary file 3 [file Table1.docx]

**Supplementary Table S1. Primers used for qRT-PCR**

| **Target** | **Accession No.** | **Species** | **Primer type** | **Sequence 5′→3′** |
| --- | --- | --- | --- | --- |
| **MALAT1** | NR_002847.3 | Rat | Forward | GTGATGCGAGTTGTTCTCCG |
|  |  |  | Reverse | CTGGCTGCCTCAATGCCTAC |
| **Bnip3** | NM_053420 | Rat | Forward | ATTGGTCAAGTCGGCCAGAA |
|  |  |  | Reverse | AGTCGCTGTACGCTTTGGGT |
| **β-actin / Actb** | NM_031144.3 | Rat | Forward | AGCCATGTACGTAGCCATCC |
|  |  |  | Reverse | CTCTCAGCTGTGGTGGTGAA |
| **miR-145-5p** | MIMAT0000851 | Rat | Stem-loop RT primer | GTCGTATCCAGTGCAGGGTCCGAGGTATTCGCACTGGATACGACAGGGAT |
| **miR-145-5p** | MIMAT0000851 | Rat | Forward | GTCCAGTTTTCCCAGGAATCC |
|  |  |  | Universal reverse | GTGCAGGGTCCGAGGT |
| **U6 snRNA** | NA | Rat | Forward | CTCGCTTCGGCAGCACA |
|  |  |  | Reverse | AACGCTTCACGAATTTGCGT |

Primer sequences are shown in the 5′ to 3′ direction. RefSeq accession numbers are provided for MALAT1, Bnip3, and Actb; the miRBase accession number is provided for miR-145-5p. NA indicates that the U6 endogenous-control primer set was not assigned to a unique RefSeq transcript.

**Supplementary Table S2.** Coagulation profiles across experimental groups.

| Variable | NC (n=4) | HS (n=8) | CFR (n=8) | RFR (n=8) | SEC (n=8) | Kruskal-Wallis H (P) | CFR vs RFR P_adj |
| --- | --- | --- | --- | --- | --- | --- | --- |
| PT (s) | 14.55 (14.02-14.95) | 18.70 (11.38-20.52) | 23.45 (21.80-27.38) | 14.70 (13.80-15.88) | 11.55 (10.97-15.20) | 17.70 (0.0014) | 0.034 |
| TT (s) | 16.80 (16.18-17.52) | 23.90 (22.58-26.75) | 29.50 (24.78-33.65) | 19.35 (18.25-19.70) | 24.55 (23.20-25.90) | 21.67 (0.00023) | 0.008 |
| APTT (s) | 20.95 (20.90-22.20) | 63.65 (59.00-66.47) | 55.45 (39.60-75.53) | 38.05 (36.45-39.62) | 16.55 (15.68-24.38) | 26.80 (<0.0001) | 0.422 |
| INR | 1.03 (1.01-1.06) | 0.81 (0.74-0.96) | 2.17 (2.10-2.26) | 1.26 (1.21-1.30) | 0.93 (0.87-1.21) | 21.23 (0.00029) | 0.395 |
| PA (%) | 95.00 (91.25-98.50) | 132.00 (123.00-134.50) | 43.00 (38.75-63.50) | 89.00 (86.75-90.25) | 135.50 (87.00-144.50) | 20.70 (0.00036) | 0.289 |

Data are median (IQR). Overall P values are from Kruskal-Wallis tests; pairwise CFR-versus-RFR P values are from Dunn tests with Holm adjustment across the 10 pairwise comparisons within each endpoint. PT, prothrombin time; TT, thrombin time; APTT, activated partial thromboplastin time; INR, international normalized ratio; PA, prothrombin activity.
